# Advancing a patient-centered and holistic approach for older patients with frailty in a vascular surgical department: a quality improvement initiative

**DOI:** 10.1007/s41999-025-01355-0

**Published:** 2025-11-28

**Authors:** Ane Borgbjerg Verholt, Catherine Hauerslev Foss, Lajla Malene Hinrichsen, Line Hauschildt Madsen, Caroline S. N. Odderskov, Lene Holst Andersen, Lone Winther Lietzen

**Affiliations:** 1 Department of Geriatrics, Aarhus University Hospital, Aarhus, Denmark; 2 Department of Quality, Aarhus University Hospital, Aarhus, Denmark; 3 Department of Vascular Surgery, Aarhus University Hospital, Aarhus, Denmark; 4 Department of Medicine, Randers Regional Hospital, Randers, Denmark; 5 Department of Clinical Medicine, Aarhus University, Aarhus, Denmark

**Keywords:** Quality Improvement, Comprehensive Geriatric Assessment, Interdisciplinary collaboration, Frail older adults, Healthcare quality, Geriatric task force, Implementation, Workplace-based learning, Complex intervention**s**

## Abstract

**Background:**

Older patients with frailty and complex needs require patient-centered care. This may challenge non-geriatric staff lacking the expertise to care for these patients. Geriatric Task Force (GTF) is a Quality Improvement initiative developed to integrate geriatric principles into non-geriatric departments through interdisciplinary workplace-based learning, using Comprehensive Geriatric Assessment (CGA) as a cornerstone for the intervention.

**Objective:**

To evaluate the implementation of the GTF initiative in a vascular surgical department (VSD).

**Methods:**

The intervention utilized the Model of Improvement and followed premises for workplace-based learning to implement a structured four-phase model: preparation, analysis and planning, implementation, and evaluation during a one-year period. Staff received CGA principles training through e-learning and supervision. Identified focus areas included identification of frailty using Clinical Frailty Scale (CFS), registration of treatment plan for resuscitation and intensive care, and the use of early discharge checklists. Evaluation measures included frequency and quality of Standardized Field Input (SFI), medical record audits, surveys, and semi-structured interviews.

**Results:**

CFS was integrated into daily routines in the ward. Registered treatment plan for resuscitation and intensive care was well implemented, with 83% of SFIs rated as high quality, whereas the preparation of discharge checklists achieved moderate adoption, 44% rating as high quality. Staff and management reported improved frailty recognition and advanced holistic care approach.

**Conclusion:**

The GTF initiative embedded geriatric principles into VSD, improving patient-centered care for older patients with frailty. While challenges remain, the intervention demonstrates potential for sustainable improvements in non-geriatric departments. Tailored approaches and strong collaboration are essential for successful implementation.

**Supplementary Information:**

The online version contains supplementary material available at 10.1007/s41999-025-01355-0.

## Key summary points

**Aim:**

To evaluate the implementation of a quality improvement initiative integrating geriatric principles of care into a non-geriatric department.

**Findings:**

integrating geriatric principles of care into non-geriatric clinical environments can be achieved using the improvement model and workplace-based learning. In a Vascular Surgical Department, identified focus areas included identification of frailty using the Clinical Frailty Scale (CFS), registration of treatment plan for resuscitation and intensive care, and the use of early discharge checklists. Engagement from clinicians and leaders is crucial when aiming to change clinical practise.

**Message:**

The Geriatric Task Force initiative offers a promising approach to meet the complex needs of the increasing population of older patients with frailty.

**Supplementary Information:**

The online version contains supplementary material available at 10.1007/s41999-025-01355-0.

## Introduction

Hospitalized older patients with frailty often present with complex health needs, including multimorbidity, polypharmacy, and social vulnerabilities [1, 2]. These patients face high risks of adverse outcomes, such as medication errors, functional decline, prolonged hospital stays, death, fragmented patient trajectories, and lack of perceived patient-centeredness [1, 3–6]. Additionally, interdisciplinary and cross-sectoral coordination during hospital stays and at discharge is essential to mitigate these risks [7]. Patients with frailty usually rely heavily on informal caregivers to support them during hospital admission and discharge. Despite this, both patients and their informal caregivers frequently report a lack of involvement and patient-centeredness, thus advocating for a cohesive and participatory patient journey [8, 9]. Involvement of patients and their relatives based on a holistic approach can reduce the negative consequences of hospital admissions and ensure an appropriate use of resources in the healthcare system [10]. These challenges may be addressed using the Comprehensive Geriatric Assessment (CGA), an evidence-based, multidisciplinary approach identifying medical, functional, and psychosocial limitations in older adults. CGA facilitates the development of individualized care plans, promoting better coordination and improving patient outcomes [11, 12].

Like many other countries, the Danish secondary healthcare sector faces increasing demands, including a growing population of older people with frailty, limited hospital bed capacity, health care workforce shortage, and pressures for economic efficiency [13]. Although only about 2% of doctors in Danish hospitals are geriatricians, the need for geriatric expertise is increasing. This disparity presents significant challenges in the provision of comprehensive care for older adults with frailty, particularly in highly specialized hospitals such as Aarhus University Hospital (AUH) where patients require both specialized treatment and a holistic approach [14],.

To address these challenges, the geriatric department at AUH developed the Geriatric Task Force (GTF) in 2021, a quality improvement (QI) initiative focused on enhancing the care of older inpatients with frailty in non-geriatric departments. The GTF follows the principles of CGA and, as such, comprises interdisciplinary geriatric staff, possessing high levels of geriatric expertise and competencies. Driven by local needs assessments focusing on identifying skill gaps and fostering organizational change, GTF provides targeted training for healthcare providers in non-geriatric departments to improve their competencies in managing frailty and ensuring coherent, patient-centered care.

The care of older patients with frailty presents unique challenges, particularly in non-geriatric settings. Recognizing these challenges, this paper aims to evaluate the GTF initiative within a vascular surgical department (VSD). By examining the impact of this initiative, we seek to contribute to the growing need of integrating geriatric principles into non-geriatric clinical environments.

## Methods

This study evaluated the introduction and impact of the GTF initiative in a non-geriatric department through quantitative and qualitative data. The intervention took place between January and November 2024. This report adheres to SQUIRE 2.0 reporting guidelines [15].

### Context

The Danish healthcare system is primarily publicly funded, with secondary care mainly provided by public hospitals. Each citizen in Denmark is assigned a unique Civil Personal Registration (CPR) number, enabling efficient patient identification and data tracking throughout the healthcare system [16]. The Clinical Frailty Scale (CFS) is a widely accepted tool currently being implemented in the Danish healthcare system for assessing frailty level of older people two weeks prior to hospitalization [17]. At AUH, an 858-bed teaching hospital, non-geriatric departments faced significant challenges in managing older patients with frailty, including prolonged hospital stays, fragmented cross-sectoral care pathway transitions, frequent readmissions, and adverse events. To address these issues, the Geriatric Department established the GTF initiative, prioritizing non-geriatric departments for participation based on criteria, such as patient demographics, average length of stay, and the department’s average length of stay, motivation, and leadership commitment. The Vascular Surgical Department (VSD) was chosen for its patient population, strong leadership commitment, and history of successful QI efforts. Patients in the Vascular Surgical Department are admitted on an acute, sub-acute, or elective basis. The department faces increasing challenges due to an aging population with multimorbidity and complex care needs.

### Geriatric task force (GTF) initiative

GTF is grounded in the model for improvement [18] and John Kotter’s eight-step model for leading organizational change [19]. Stephen Billett’s four premises for workplace-based learning [20] provide a foundational understanding of the initiative in a hospital setting by highlighting 1) learning takes place at work; 2) engagement from workers transform occupational activities; 3) culture and physical aspects shape clinical knowledge; and 4) learning and development are different but interdependent processes [21]. The GTF initiative was presented to a patient panel from Aarhus Municipality’s Senior Citizens’ Council during its development and received unanimous support from both patients and informal caregivers [22].

The GTF intervention has developed over time to unfold over four key phases, designed to be universally applicable in non-geriatric departments. These phases—Preparation, Analysis and Planning, Implementation, and Evaluation—are described in Table 1.

**Table 1:** Overview of the four phases in a Geriatric Task Force visit to a non-geriatric department

	Phase 1 preparation	Phase 2 analysis & planning	Phase 3 intervention	Phase 4 evaluation & follow-up
Duration	1–3 Months	1 Month	4–5 Months	2–6 Months
Purpose	Align goals and expectations	Identify focus areas for improvement Build relationship between the non-geriatric department staff and GTF Ensure shared understanding of the department’s clinical work, culture, and patient population	Implement chosen focus areas GTF staff supporting the non-geriatric department through workplace-based learning	Evaluate to ensure ongoing maintenance of initiated initiatives Highlight possible areas for continued development
Key activities	Collaboration between Geriatric and non-geriatric department managements with GTF to ensure management support Formation of a quality improvement team with commitment and the ability to lead improvement initiatives Conducting introductory meetings with staff, including a presentation of GTF's purpose, methods, and expectations. An opportunity for all staff to describe the challenges they face when working with older patients with frailty Staff completing an e-learning program concerning older patients with frailty Meeting coordination and planning	Presence of GTF in the non-geriatric department twice weekly and engaging in the daily operations of patients in the department Workflow analysis of common patient pathways, using the CGA as framework GTF and local improvement team decide focus areas Developing driver diagrams to identify key factors influencing project outcomes and to guide the implementation strategy	Presence of GTF in the non-geriatric department twice weekly to provide coaching, supervision, and formal education Tracking improvements using Plan-Do-Study-Act (PDSA) cycles to implement, evaluate, and continuously refine changes Follow data to ensure continuously progress Establish a data wall in the department where staff can stay updated on ongoing initiatives and data	Producing a plan for sustaining and advancing the initiatives Providing a final report with observations, interventions, results, and recommendations The management of the non-geriatric department is responsible for maintaining improvements and implementing recommendations, with GTF available for guidance and advice GTF re-visit the non-geriatric department after 2–4 months to evaluate the collaboration and support anchoring and further development
Data	Baseline data on the patient population in the non-geriatric department	Workflow analysis Driver diagram	PDSA cycles SFI utilization in run charts Journal audits	SFI utilization series diagrams Journal audits Staff questionnaires Semi-structured interviews with non-geriatric department management
Case	January–Marts 2024	April 2024	May–September 2024	October–November 2024
Vascular surgical department (VSD)	Management collaboration between the Geriatric Department and VSD managements Forming the improvement team of a geriatric senior consultant and physiotherapist (GTF) and a senior consultant, consultant, and head nurse (VSD) Identification of challenges with treatment of older patients Establishing a shared understanding of frailty and CGA through e-learning developed by GTF team Planning of progress meetings every three weeks with the improvement team and every eight weeks with departmental management	Participation of GTF in VSD outpatient clinic, surgical procedures, and ward rounds Performing workflow analysis by the improvement team and additional staff representatives and setting the focus areas of the intervention: 1. Enhancing frailty recognition 2. Establishing agreed treatment levels with critical care and resuscitation 3. Preparation for early discharge	The Clinical Frailty Scale (CFS) was introduced to all staff through interdisciplinary teaching, an e-learning program and assessment materials Posters were displayed in the department Patients aged ≥ 65 were assessed using CFS, with Standardized Field Inputs (SFIs) completed under GTF supervision GTF provided training on treatment levels, critical care discussions, coaching, and case-based education to improve communication Nursing staff were trained in discharge procedures and developed a standard text and checklist for patients with CFS ≥ 4 A driver diagram guided the intervention (Fig. 3) PDSA cycles tracked progress, and run charts with SFIs and medical record audits monitored outcomes	Final meetings with agreement on sustaining the implementation of CFS and treatment levels, further utilizing discharge checklists, and identifying other areas for development Planning two-month evaluation of the project and ongoing initiatives, while VSD was given the opportunity to contact GTF for further guidance and advice VSD proposed a network for departments using GTF to share experiences and support further development Evaluation was conducted via questionnaire (Appendix 1) and semi-structured interviews (Appendix 2) Producing final report of process insights and recommendations for sustaining and improving initiatives

Generical and exemplified by the case of Vascular Surgical Department at Aarhus University Hospital, Denmark

*GTF* Geriatric Task Force, *PDSA Cycles* Plan-Do-Study-Act Cycles, *VSD* Vascular Surgical Department, *CGA* Comprehensive Geriatric Assessment, *CFS* Clinical Frailty Scale

### Measures

In Phase 2, the improvement team decided on three focus areas for the GTF intervention: enhancing frailty recognition, establishing agreed treatment levels with critical care and resuscitation, and preparing early discharge (Table 1). Quantitative and qualitative data assessed VDS staff improved their competencies in these focus areas.

To provide structured documentation for run charts,* quantitative* data on process and balancing indicators were collected from the Electronic Health Record (EHR) using Standardized Field Inputs (SFIs). SFIs provide a predefined and structured documentation tool (see Appendix 1), including sub-items costumed to a given clinical task. The SFI can both support the clinician to remember the task and provide a structured possibility to document findings and decisions such as DNRS or discharge planning. Process indicators reflected the proportion of completed SFIs on CFS (outpatient clinic and ward), treatment plan for resuscitation and intensive care (outpatient clinic and ward), and discharge checklists (ward). To assess unintended consequences due to the intervention, 30-day readmissions were monitored as a balancing indicator [23]. The SFI quality was assessed through medical record audits of 42 EHRs by GTF members evaluating the documentation against predefined criteria, such as patient involvement and consistent documentation of sub-items line by line. The findings were presented in bar charts, serving as a basis for targeted feedback and further refinement of documentation practices. Data were analyzed using series diagrams and Statistical Process Control (SPC) run charts to monitor changes [18].

*Qualitative* feedback was gathered through staff surveys and semi-structured interviews [18]. Surveys, including open-ended questions, evaluated the perceptions of the intervention, the effectiveness of training, and its overall impact (the survey questions appear in Appendix 2). The staff survey was developed by a quality consultant from the hospital’s Quality Department with prior experience in designing employee questionnaires. It was created in collaboration with clinicians and underwent pilot testing among staff from VSD before its final distribution. Semi-structured interviews with departmental management were conducted by the quality consultant, to get feedback on intervention effectiveness and identified challenges (interview guide appears in Appendix 3). Thematic analysis identified common themes and insights [24].

### Ethical considerations

Due to the nature of QI projects, formal approval from data protection agencies and individual consent from participants were not required. Access to EHR data was approved by hospital management.

The CFS was employed as a decision-support tool rather than a definitive determinant of patient care, ensuring careful attention to ethical considerations in treatment decisions rather than introducing frailism when deciding care pathways [25].

## Results

The GTF intervention evaluation included quantitative and qualitative measures to assess impact on processes and outcomes in the VSD.

### Quantitative data

During the intervention period, an average of 63 inpatients aged 65 years or older were admitted to the ward per month, and approximately 300 outpatients aged 65 years or older were seen in the clinic per month. All patients over 65 years were intended to be assessed using the CFS and to have agreed the treatment levels documented. Early discharge planning was provided only for admitted inpatients.

**Fig. 1:**
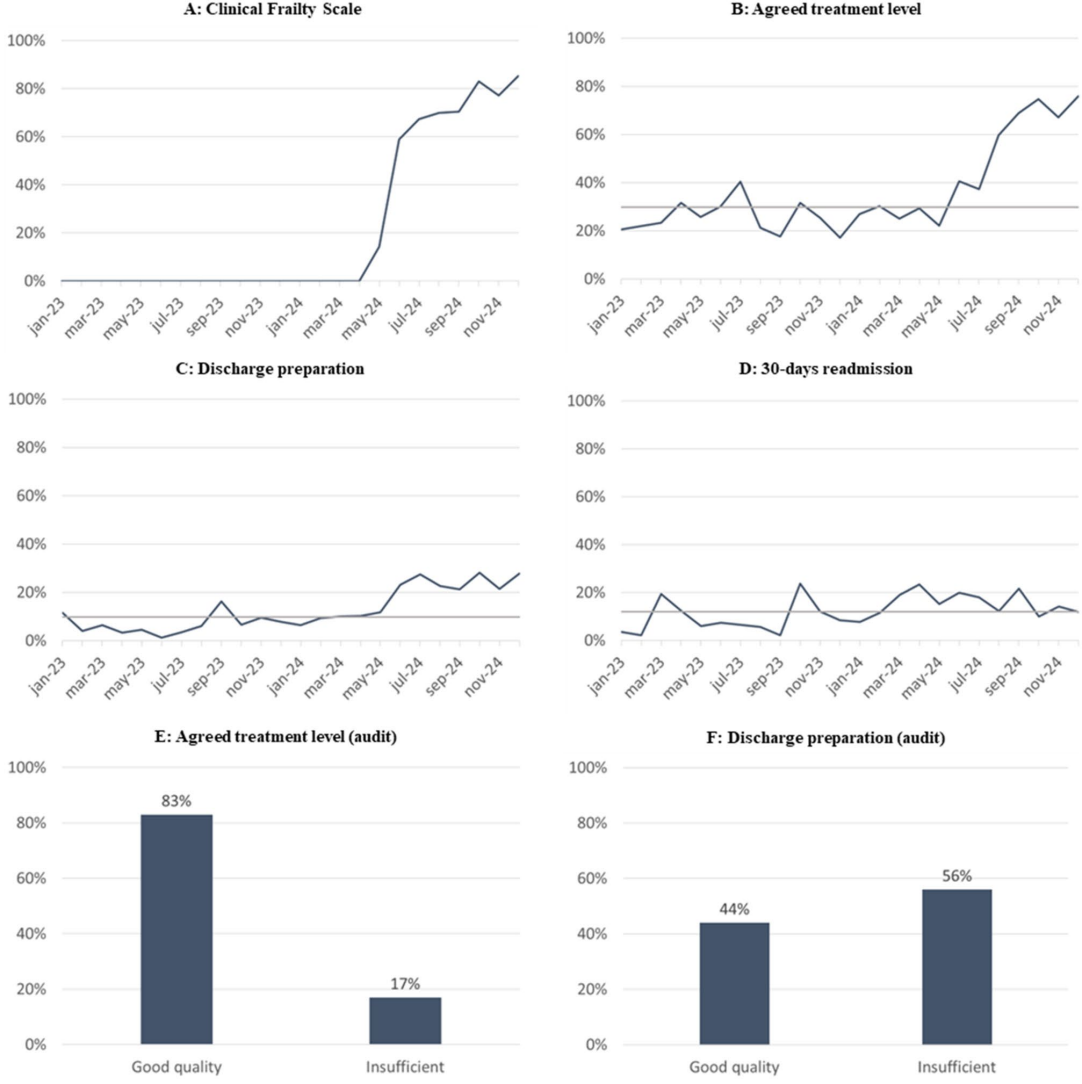
Examples of run charts and bar charts from Vascular Surgical Department. Percentage of patients $\ge$ 65 years with completed Standardized Field Inputs (SFIs) (**a**-**d**) and the quality of the text in the SFI (E–F). **a** Completion of SFI with “Clinical Frailty Score” (CFS)$\ge$ 65 years. **b** Completion of SFI with “Agreed treatment level” among patients $\ge$ 65 years. **c** Completion of SFI with “Discharge preparation” among patients $\ge$ 65 years. The SFIs were used as proxy for discharge preparation for older patients with frailty, as they were sent to the municipality nurses. **d** Readmissions among patients $\ge$ 65 years. Balance indicator to make sure that interventions did not cause unintended consequences. **e** Quality of SFI text for “Agreed treatment level” in patients with CFS $\ge$ 4 assessed in audit. **f** Quality of SFI text for “Discharge preparation” in patients with CFS $\ge$ 4 assessed in audit. Four run charts describing the percentage in completed Standardized Field Inputs by time and two bar charts describing the quality of text in Standardized Field Inputs from audit

Figure 1 presents ward-level data. For both outpatients and hospitalized patients, CFS implementation showed a clear, non-random pattern of variation. Run chart for treatment plan for resuscitation and intensive care SFIs are also shown. In the outpatient clinic, only normal variation was seen, whereas hospitalized patients in VSD showed non-random variation. A data quality audit showed that 83% of completed SFIs were of good quality. For discharge preparation, SFIs completeness data showed non-random variation, and 44% were of good quality. No increase in readmissions was observed in VSD during the study period.

### Qualitative data

**Fig. 2:**
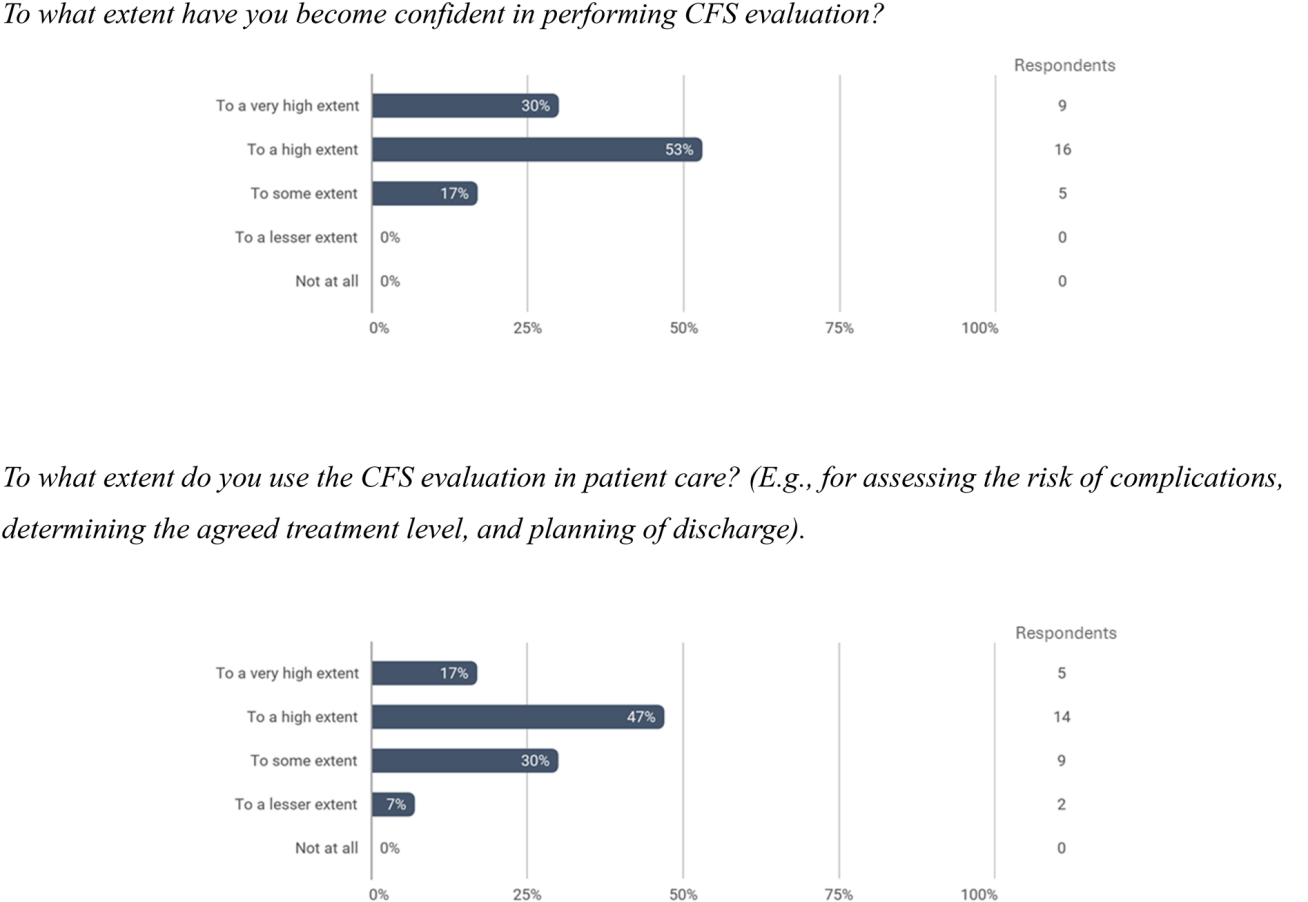
Examples from Vascular Surgical Department qualitative data. Full questionnaire is presented in Appendix 1. Questionnaire filled in by staff: *To what extent have you become confident in performing CFS evaluation? To what extent do you use the CFS evaluation in patient care? (E.g., for assessing the risk of complications, determining the agreed treatment level, and planning of discharge).* Bar chart examples of answers from questionnaire filled in by staff members. The bars indicate that 83% of staff feel confident in performing CFS evaluation to a high or very high extent, and that 64% of staff use the CFS evaluation in patient care to a high or very high extent

Figure 2 and Table 2 present data from staff and management surveys and interviews. In total, all nurses and physicians from the VSD were involved in the project (*n* = 39) and invited to participate in the survey. Of these, 30 completed it (response rate 77%). All 39 staff members participated in the training and intervention to varying degrees*.* Most staff members reported high confidence in performing CFS evaluations, with 83% indicating confidence to a high or very high extent. The majority also reported to use the CFS in patient care, 64% to a high or very high extent and 30% to some extent. Thematic analysis resulted in three themes: Common language and structure, Structured process with focus on the department's needs, and Organizational significance. Quotes from interview and questionnaire are cited in Table 2 and quotes from leader interview are interpreted based on Kotter’s 8 steps of change in Appendix 4.

**Table 2:**
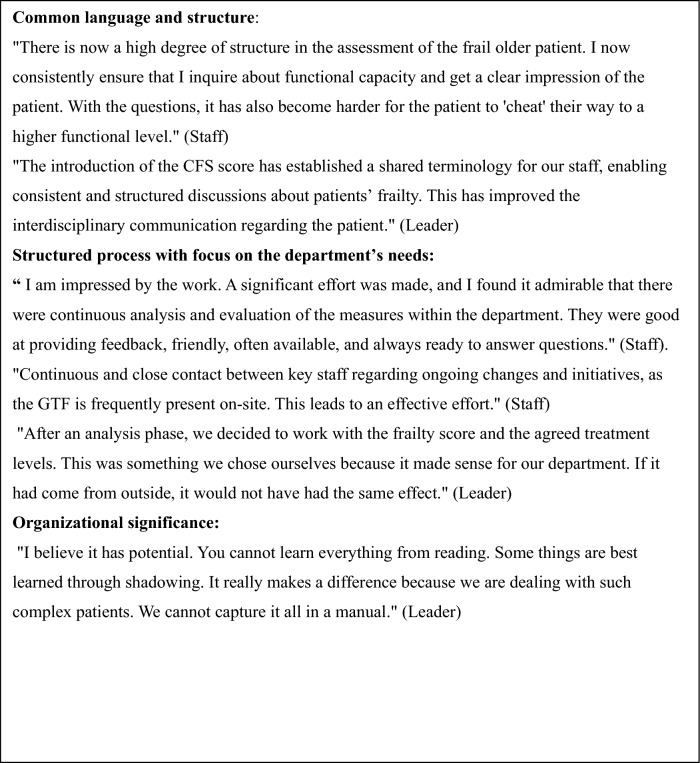
Examples from Vascular Surgical Department qualitative data Thematic mapping of leadership and staff comments based on data from leader interview and free text in questionnaire. The full interview guide is presented in Appendix 2

## Discussion

This study demonstrates a QI initiative aimed at enhancing care for older patients with frailty exemplified by an intervention at a VSD. As a workplace-based intervention, the GTF embeds geriatric principles into non-geriatric departments by fostering interdisciplinary collaboration and patient-centered care. The findings from this study illustrate the practical implementation of the initiative, offering insights into its impact and the challenges encountered during its execution. The VSD experienced a change in daily routines supporting a holistic approach toward older patients with frailty. The recognition of frailty was fully integrated into the ward and served as the basis for further implementation, whereas the implementation of a treatment plan for resuscitation and intensive care proved challenges and required particular attention. Early discharge checklists required continued reinforcement to ensure consistent use and high quality. The choice of this focus area aligned with perceived need among staff during the workflow analysis and was consistent with moderate-certainty evidence in the literature demonstrating lower readmission rates and reduced the length of stay when delivered as a structured, multidisciplinary, and patient-centered intervention [26, 27].

**Fig. 3:**
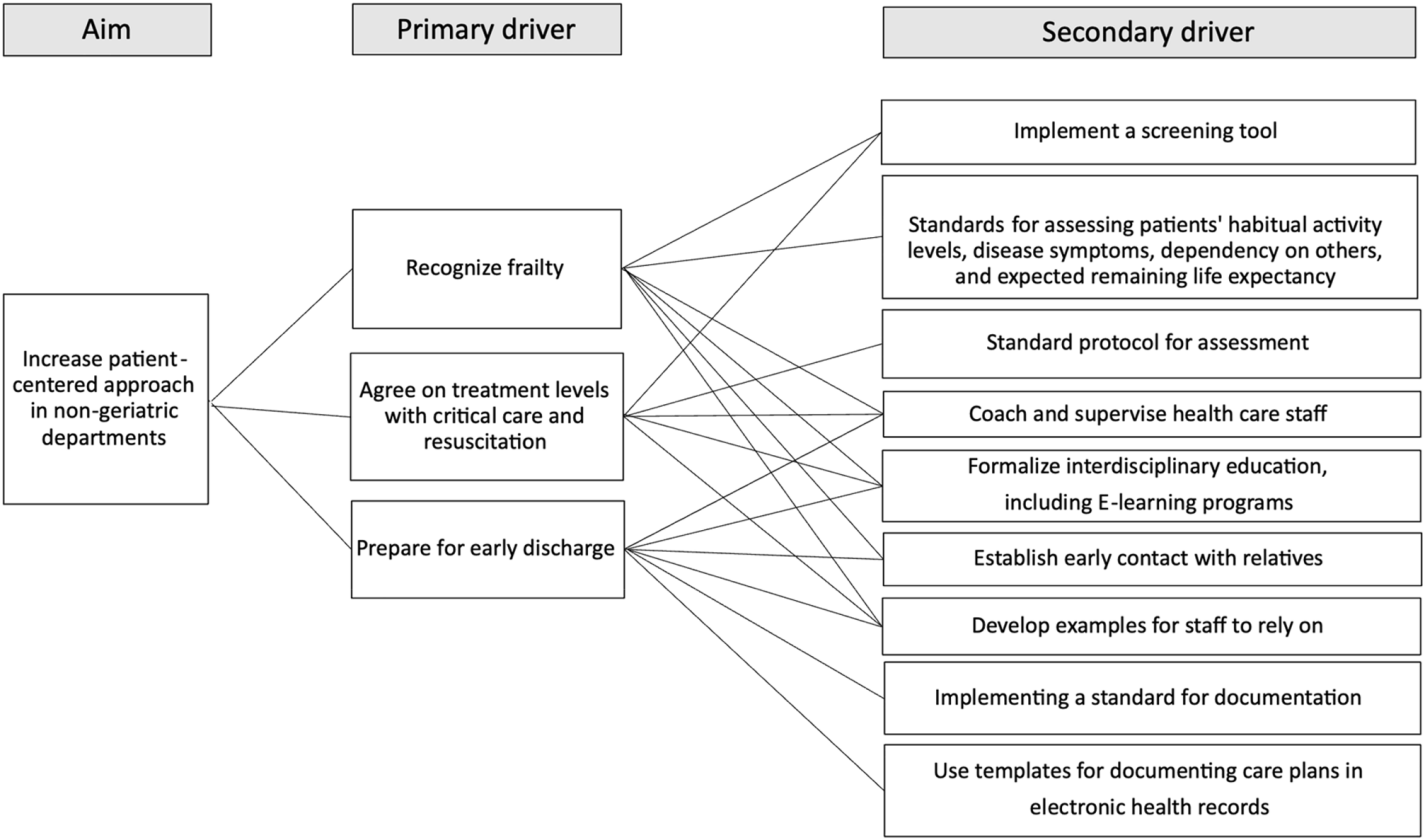
Example of a driver diagram in the Vascular Surgery Department. Driver diagram showing the Aim (increase patient-centered approach in non-geriatric departments), three primary drivers (recognize frailty, agree on treatment level, and early discharge preparation), and nine secondary drivers to support the change

Both staff and management reported that the implemented changes led to significant improvements and that the time invested was justified. These findings underscore the importance of structured processes, tailored support, and active engagement from staff in achieving sustainable change (Fig. 3).

There are several examples of established binding of specific medical and/or surgical disciplines to geriatric co-management, including ortho-geriatrics [28], onco-geriatrics [29], and geri-cardiology [30]. However, the focus of the geriatric task force is particularly on creating a learning organization rather than co-management. The geriatric task force approach can strengthen and support existing collaborative structures and should not be seen as an either/or solution. However, even well-designed collaborative models rely on effective change management to be successfully implemented.

Attention to Kotter’s change management steps and potential pitfalls is very important when analyzing reasons for implementation challenges [19, 31]. In this study, establishing treatment plans for resuscitation and intensive care proved challenging. Similar to other findings, doctors struggled to engage in these discussions, often perceiving the need for change as non-urgent, leading to prolonged implementation periods and necessitating adjustments in communication strategies [32, 33]. These challenges align with challenges in the initial steps in Kotter's change management framework. Step 1, creating a sense of urgency, was often lacking in the outpatient clinic but emerged on the ward prior to surgery, where doctors and nurses saw these discussions as crucial due to potential life-threatening events in the operating room. The workflow analysis and decisions reinforced shared purpose among staff. This impact could have been stronger if Steps 2 and 3—building a coalition and developing a clear strategy—had been implemented earlier, aligning with literature advocating all eight steps for successful change [34, 35]. The challenge in creating urgency among staff highlights the need for tailored communication strategies linking a change to patient outcomes. Variations in readiness, attitude, and engagement across units and departments suggest that a one-size-fits-all approach may not be effective; rather, interventions should be adapted to meet the specific needs and contexts of each department.

Discharge preparation in VSD using a standardized checklist for patients with a CFS score ≥ 4 resonated well with the staff although they required reminders and support to complete it effectively, aligning with Step 2, building a guiding coalition. According to a review by Rameli and Rajendran [36], frailty is a main course for delayed discharge and should guide complex discharge planning from admission start to permit staff to anticipate and overcome potential barriers to discharge. The GTF recognized that practices like discharge planning—routine for geriatric staff—may be more challenging for non-geriatric departments. Assumptions of shared understanding may cause misunderstandings, underscoring the need for clear expectations.

The CFS served as a decision-support tool, clarifying the rationale behind treatment decisions and ensuring patient-centerdness. CFS became a common language across disciplines and provided a foundation for implementation. Other studies have shown that CFS or similar frailty assessments may predict patient outcomes [37, 38]. Furthermore, higher CFS scores are associated with longer hospital stays and increased 30-day readmission rates [39]. Thus, utilizing the CFS $\ge$ 4 to select patient for early discharge preparation could mitigate these risks, as suggested by other studies [38, 40].

Defining treatment levels for resuscitation and intensive care is a key component of Advance Care Planning, helping to align care with patient preferences and potentially to avoid non-beneficial interventions [41].

### Workplace-based learning, sustainability, and broader implications

Other studies have experienced difficulties in embedding CGA initiatives in non-geriatric services, and the prior engagement of VSD in patient-centered practices facilitated the simultaneous initiation of multiple initiatives [42, 43]. As such, when considering the context in which GTF operates, it is important to recognize that establishing fundamental elements of patient-centered practice is often a prerequisite before other initiatives can be successfully implemented.

The insights derived from GTF align with Billett’s four premises for workplace-based learning, emphasizing that refining procedures and clinical reasoning are achievable through structured and supported routine clinical activities [20]. This alignment is most evident when clinicians are actively engaged, share common and professional goals, and operate within a department fostering a culture of change and receiving support from leadership [44, 45].

The results of the intervention were driven by strong management, a collaborative culture, and effective teamwork. The four phases of the intervention have defined purposes that support a shared understanding of the process between GTF and the non-geriatric departments.

To effectively disseminate GTF's approach to teaching geriatrics in non-geriatric departments, geriatric interdisciplinary specialists must demonstrate their expertise and extend their services beyond their specialties. Non-geriatric departments, in turn, must recognize their responsibility to provide patient-centered, holistic care for older adults with frailty and multimorbidity. This requires a commitment to enhancing competencies for this growing patient group, which, despite historically receiving less attention, now represents a significant healthcare demand [46]. To support this, management and policymakers should prioritize the needs of this population at all levels [47]. A study by Meranius et al. (2017) identified seven core components essential for effective health and social care management of older adults with multimorbidity, including competence, collaboration, and political steering [48]. Similar, during an interview with VSD management, it was suggested that departments receiving GTF interventions form local networks to share experiences and ideas, strengthening a holistic approach to older patients with frailty and inspiring future improvements, independent of the Department of Geriatrics [42].

The strengths of the QI initiative are that the intervention was conducted in a real-life hospital setting and with interprofessional staff members involved, thereby enhancing the practical relevance and applicability of the findings. Outcome accuracy was assured through the utilization of a comprehensive range of valid and reliable data sources, including EHRs, medical record audits, and qualitative interviews and surveys. By integrating both qualitative and quantitative methods, the project provided a comprehensive and balanced assessment of the impact of the interventions [31, 49].

This project has several limitations that may affect the generalisability of its findings. Specifically, the results may have limited external validity due to the unique context of AUH and the Danish Healthcare System. The distinctive organizational structures, cultural dynamics, patient demographics, and healthcare policies in this region may not be directly applicable to other healthcare settings. Also, no data was collected on patient satisfaction or whether the primary sector experienced improvements in the communication because of the initiative. Further, no patient-level clinical outcomes were measured. The focus was on process-related changes, and on targeting departmental needs using validated tools to optimize workflows. Absence of direct patient-level outcomes, such as patient satisfaction, quality-of-life, readmission rates, and mortality, was a limitation of the study and would have further strengthened the results. In future studies, insights from stakeholders could provide a more comprehensive understanding of the impact of the initiative on both patients, relatives, and collaborators in the primary care sector. However, some elements of our approach could be transferable to other hospitals facing similar challenges. Factors that may have compromised the internal validity of the study include potential confounding variables, such as competing improvement projects and the prioritization of other activities, as well as information biases, and imprecision in the intervention design and implementation. To mitigate the impact of these challenges, we employed multiple evaluation methods, ensuring rigorous data collection and applying the interventions in real-life settings [49].

## Conclusion and perspectives

The GTF initiative promotes a patient-centered, holistic approach in non-geriatric departments. We evaluated the implementation of the GTF initiative as a QI project in a Danish surgical department. By encouraging interdisciplinary workplace-based learning and collaboration, GTF addressed the complex health needs of older patients with frailty, demonstrating the value of integrating geriatric principles into diverse clinical settings. It highlighted the need for a coordinated approach across hospital departments and provided a sustainable model for improving care delivery. GTF enhanced healthcare providers' competencies, such as recognizing frailty, agreeing on treatment level, and delivering high-quality early discharge plans. GTF may offer a framework for other facilities facing similar challenges. As a model for patient-centered care, it emphasizes prioritizing the needs of vulnerable populations within the healthcare system.

The GTF intervention aims to promote a sustainable methodology that addresses the challenges posed by the increasing number of older patients with frailty amid resource constraints. It is expected to enhance patient-centered and holistic approaches while driving procedural improvements in workload management, resource allocation, and patient pathways in non-geriatric departments. By fostering a common language and clear care plans, the GTF intervention is anticipated to have a positive impact on future patient care pathways, including smoother transitions to primary care.

To build on the GTF model, political recognition is needed to address challenges, such as the aging population, the shortage of geriatric specialists, and the demand for comprehensive care strategies. Ongoing investment in geriatric medicine and specialized training is essential for sustaining progress. Future research should focus on refining and expanding the GTF model to additional healthcare settings, ensuring continuous adaptation to the needs of older patients with frailty, and improving their hospital experiences and care pathways. Finally, longitudinal assessments of patients’ trajectories, guided by the insights from the GTF initiative, could potentially transform clinical practice to better accommodate the needs of patients and their relatives.

## Supplementary Information

Below is the link to the electronic supplementary material.

Supplementary file1 (DOCX 90 KB)

## Data Availability

Data are available upon reasonable request.
