# Supplementary material for: Advancing a patient-centered and holistic approach for older patients with frailty in a vascular surgical department: a quality improvement initiative
Source: Eur Geriatr Med. 2025 Nov 28;17(2):837–49. doi: 10.1007/s41999-025-01355-0 (PMC13109110; doi:10.1007/s41999-025-01355-0)
Supplement: Supplementary file 1 — Supplementary file1 (DOCX 90 KB) [file 41999_2025_1355_MOESM1_ESM.docx]

# Supplementary Information

### Title: Appendices for: Advancing a patient-Centered and holistic approach for older Patients with frailty in a vascular surgical department;:a quality improvement initiative

Submitted to Journal of European Geriatric Medicine
Ane Borgbjerg Verholt^1^ ORCHID: 0000-0002-0482-9129

Catherine Hauerslev Foss^1^

Lajla Malene Hinrichsen^1^ ORCHID: 0009-0009-9896-7474

Line Hauschildt Madsen^2^

Caroline S.N Odderskov^3^ ORCHID: 0009-0009-4703-7955

Lene Holst Andersen ^1,4,5^ ORCHID: 0000-0003-0331-3136

Lone Winther Lietzen^1,5^ ORCHID: 0000-0002-9520-406X Corresponding author lwl@clin.au.dk

^1)^ Department of Geriatrics, Aarhus University Hospital, Aarhus, Denmark

^2)^ Department of Quality, Aarhus University Hospital, Denmark
^3)^ Department of Vascular Surgery, Aarhus University Hospital
^4)^ Department of Medicine, Randers Regional Hospital, Randers, Denmark
^5)^ Department of Clinical Medicine, Aarhus University, Aarhus, Denmark

Appendix 1 Example of Standardized Field Inputs (SFI) for agreed treatment level as seen in the Electronic Health Record (EHR) [in Danish].

Appendix 2 Staff survey was developed and distributed to evaluate whether staff had achieved a competence boost in applying a holistic, patient-centred approach.

Appendix 3 Interview Guide for Semi-Structured Interview with the Management of the Receiving Department

Appendix 4 Quotes from leader interview interpreted based on Kotter's 8 steps of change

Appendix 1

Example of Standardized Field Input (SFI) for “agreed treatment level” as seen in the Electronic Health Record (EHR) [in Danish]. This EHR is made by Systematic in collaboration with local clinicians (<https://systematic.com/int/industries/healthcare/news/news/2020_cis_electronic-health-record-improves-patient-safety/> )

This screen dump (from a test person) illustrates the mandatory and voluntary elements to tick off, including whether it is an “agreed treatment level”, and the reason for this together with information for patients/relatives.


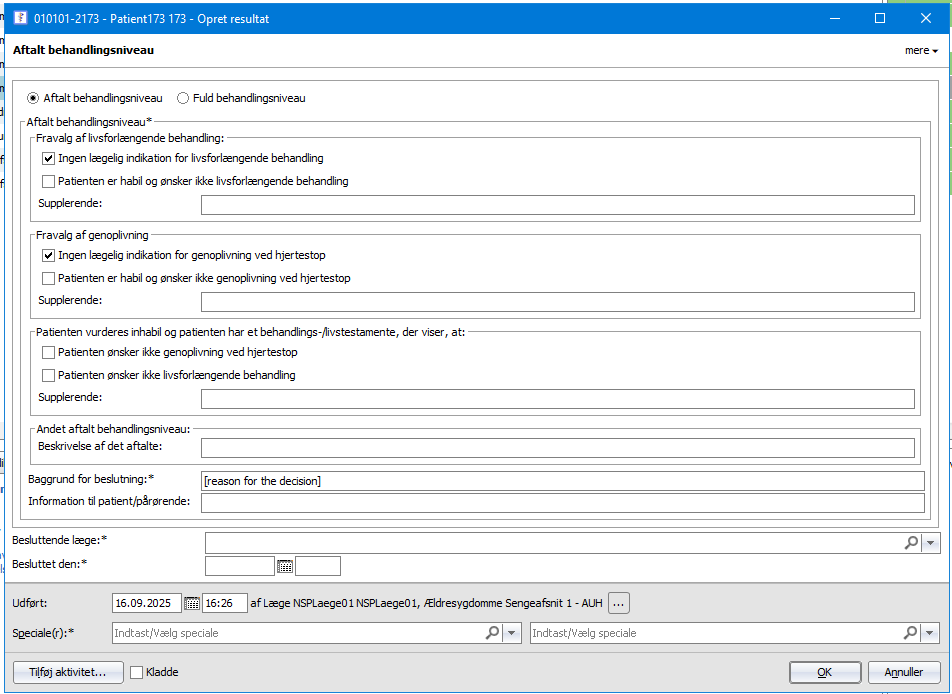


**Appendix 2:**
Staff survey was developed and distributed to evaluate whether staff had achieved a competence boost in applying a holistic, patient-centered approach.

Questions for staff survey:

**Thank you for taking the time to complete this questionnaire.**

You are receiving this questionnaire because your department has been visited by the Geriatric Task Force.

The purpose of the questionnaire is to gain insight into your experience of the visit and whether it has influenced your competencies.

It takes approximately 4–8 minutes to complete. Your response will be treated confidentially.

**Kind regards,**
Geriatric Task Force
Department of Geriatric Medicine, Aarhus University Hospital

Basic information:

**1. Which professional group do you belong to?**
(1) Doctor
(2) Nursing and care staff

During our visit to your department, we conducted various activities including side-by-side training, supervision, professional sparring, and teaching.

**2. Did you participate in one or more of these activities?**
(1) Yes
(2) No

**2.1. What was the reason you did not participate? [free text response]**

**3. At the outset, did you receive clear information regarding the purpose of the visit from the Geriatric Task Force?**
(1) Yes
(2) No

**Please elaborate on your answer: [free text response]**

In the following questions, we ask you to assess whether you have improved your competencies in several areas related to older patients with frailty.

**4. To what extent have you improved in assessing the patient’s habitual functional level?**
(e.g., knowing the patient’s usual functional level and network before the current admission or clinic consultation)
(5) To a very high extent
(4) To a high extent
(3) To some extent
(2) To a lesser extent
(1) Not at all
(99) Don’t know/Not applicable

**Please elaborate on your response:**

**5. To what extent have you improved in identifying frailty in older patients?**

(5) To a very high extent
(4) To a high extent
(3) To some extent
(2) To a lesser extent
(1) Not at all
(99) Don’t know/Not applicable

**Please elaborate on your response:**

The following questions relate to the Clinical Frailty Scale (CFS).

**6. To what extent have you become confident in assessing CFS?**
(5) To a very high extent
(4) To a high extent
(3) To some extent
(2) To a lesser extent
(1) Not at all
(99) Don’t know/Not applicable

**Please elaborate on your response:**

**7. To what extent do you use the CFS score during the patient pathway?**
(e.g., for assessing risk of complications, agreed treatment level, and discharge planning)
(5) To a very high extent
(4) To a high extent
(3) To some extent
(2) To a lesser extent
(1) Not at all
(99) Don’t know/Not applicable

**Please elaborate on your response:**

The following questions concern interdisciplinary collaboration.

**8.1. To what extent have you improved in collaborating with nursing and care staff about older patients with frailty?**
(e.g., coordinated knowledge sharing and preparation of a shared plan)
(5) To a very high extent
(4) To a high extent
(3) To some extent
(2) To a lesser extent
(1) Not at all
(99) Don’t know/Not applicable

**Please elaborate on yourresponse:**

**8.2. To what extent have you improved in collaborating with doctors about older patients with frailty?**
(e.g., coordinated knowledge sharing and preparation of a joint plan)
(5) To a very high extent
(4) To a high extent
(3) To some extent
(2) To a lesser extent
(1) Not at all
(99) Don’t know/Not applicable

**Please elaborate on your response:**

**8.3. To what extent have you improved in collaborating with therapists about older patients with frailty?**
(Physiotherapists and occupational therapists not from the Geriatric Task Force)
(e.g., coordinated knowledge sharing and preparation of a joint plan)
(5) To a very high extent
(4) To a high extent
(3) To some extent
(2) To a lesser extent
(1) Not at all
(99) Don’t know/Not applicable

**Please elaborate on your response:**

**9. To what extent have you improved in preparing the discharge of older patients with frailty in a timely manner?**
(5) To a very high extent
(4) To a high extent
(3) To some extent
(2) To a lesser extent
(1) Not at all
(99) Don’t know/Not applicable

**Please elaborate on your response:**

**10. To what extent have you improved in contributing to a holistic assessment of older patients with frailty?**
(e.g., functional assessment, nutrition, elimination, social circumstances, cognition, and somatic status including comorbidity)
(5) To a very high extent
(4) To a high extent
(3) To some extent
(2) To a lesser extent
(1) Not at all
(99) Don’t know/Not applicable

**Please elaborate on your response:**



**11. To what extent have you improved in involving the patient early in the process?**
(5) To a very high extent
(4) To a high extent
(3) To some extent
(2) To a lesser extent
(1) Not at all
(99) Don’t know/Not applicable

**Please elaborate on your response:**



**12. To what extent have you improved in involving relatives early in the process?**
(5) To a very high extent
(4) To a high extent
(3) To some extent
(2) To a lesser extent
(1) Not at all
(99) Don’t know/Not applicable

**Please elaborate on your response:**




The next questions concern SFI [Standardised Field Input] tools and documentation.

**13.1. Do you use the SFI “Discharge Checklist”?**
(1) Yes
(2) No

**13.1.2. To what extent have you improved in completing the SFI “Discharge Checklist”?**
(5) To a very high extent
(4) To a high extent
(3) To some extent
(2) To a lesser extent
(1) Not at all
(99) Don’t know/Not applicable

**Please elaborate on your response:**



**13.2. Do you use the SFI “Discharge Status”?**
(1) Yes
(2) No

**13.2.1. To what extent have you improved in completing the SFI “Discharge Status”?**

(5) To a very high extent
(4) To a high extent
(3) To some extent
(2) To a lesser extent
(1) Not at all
(99) Don’t know/Not applicable

**Please elaborate on your response:**

**13.3. Do you use care pathway plans?**
(1) Yes
(2) No



**13.3.1. To what extent have you improved in completing care pathway plans?**
(5) To a very high extent
(4) To a high extent
(3) To some extent
(2) To a lesser extent
(1) Not at all
(99) Don’t know/Not applicable

**Please elaborate on your response:**



**13.a. To what extent have you improved in clarifying the treatment level early in the course?**
(5) To a very high extent
(4) To a high extent
(3) To some extent
(2) To a lesser extent
(1) Not at all
(99) Don’t know/Not applicable

**Please elaborate on your response:**

**13.b. To what extent have you improved in ensuring accurate documentation in the SFI “Agreed Treatment Level”?**
(5) To a very high extent
(4) To a high extent
(3) To some extent
(2) To a lesser extent
(1) Not at all
(99) Don’t know/Not applicable

**Please elaborate on your response:**



Department of Geriatric Medicine has developed the e-learning course *“Frailty in Older Hospitalised Patients”*, which can be found on the regional e-learning platform.

**14. Have you completed the e-learning course *“Frailty in Older Hospitalised Patients”*?**
(1) Yes
(2) No

**14.1. In your opinion, was there a good connection between the e-learning course and the visit from the Geriatric Task Force?**
(1) Yes
(2) No

**Please elaborate on your response:**



The following final questions relate to your experience of the visit from the Geriatric Task Force.

**15. Was there anything that worked particularly well during the visit from the Geriatric Task Force?**



**16. Was there anything that worked less well during the visit from the Geriatric Task Force?**



**17. Do you have any final comments?**

**Thank you for your response!**



There are no more questions.

Click 'Finish' to close the questionnaire.

**Appendix 3**:

Interview Guide for Semi-Structured Interview with the Management of the Receiving Department

**Introduction:**
The interview concerns your experiences and reflections regarding the visit from Geriatric Task Force. *Value for money.*
Duration: 30 minutes
Recording: The interview will be audio recorded and subsequently transcribed. If desired, the transcription can be sent to you for review.

**Expectations:**

- What expectations did you have regarding the process/visit from the Geriatric Task Force?

**Impact and Outcomes:**

- What specific improvements have you observed in day-to-day practice as a result of the project?
- Have you noticed any unexpected outcomes (positive or negative) ?
- What has your department gained from the collaboration?

**Prioritisation and Resources:**

- In hindsight, do you believe the project was worth prioritising for your department?
- Were the time and resources you invested in the project well spent?

**Long-term Effects:**

- Do you believe the project will have any long-term benefits or lead to changes for the department and the organisation as a whole?
- Do you think you will need support for maintaining or further developing outcomes following the project?

**Areas for Improvement:**

- Is there anything you think could have been done differently to get even more out of the project?
- What adjustments would you suggest if the project was to be repeated?

**Conclusion:**

- Were your expectations met?
- Is there anything we have not covered that you believe is important to mention?

**Appendix 4:**

**Quotes from leader interview interpreted based on Kotter's 8 steps of change:**

| **Kotter’s step** | **Quotes** |
| --- | --- |
| **1. Create urgency:** | *"We already knew when things went wrong with the patients and that we intervened too late. We needed help to structure things and develop a shared language about who these patients are."* |
| **2. Build coalition:** | *"We have a really good interdisciplinary collaboration, and I think that this has been the key to our success. We do not consider it as either a doctor’s or a nurse’s task. It is a shared responsibility for the patient who needs to go through a care process."*    *"I donot think we could have reached this outcome if it had been handled as purely a medical or a nursing project. It was necessary for it to be a collective effort."*    *"There is also the aspect of leadership support. That is why I have pushed for it, and section leaders and stakeholders have as well."* |
| **3. Developing vision:** | *"After an analysis phase, we decided to work with the frailty score and the agreed treatment levels. This was something we chose ourselves because it made sense for our department. If it had come from outside, it wouldn’t have had the same effect."* |
| **4. Communicate the vision:** | *"The project team was really good at taking five minutes during meetings to explain why this made sense. They could put it into a broader perspective beyond just our department, making it part of something larger."* |
| **5. Empower others to act:** | *"The external input was healthy. It shook things up that would otherwise have remained the same."* |
| **6. Planning and achieving short-term results:** | *"Just having the physiotherapist here made a difference. I could quickly ask her something, and others did the same. We tried things out immediately and made adjustments the following week if needed. It is so easy when you know there is a follow-up again on Tuesday – instead of waiting for a meeting with a resource group every other month. That just does not have the same impact. Seeing progress is motivating."* |
| **7. Consolidating results and generating more change:** | *"We hold regular meetings to follow up on how things are going. This helps us ensure we do not fall back into old habits. We are in the process of integrating this into the training of new staff – both nurses and doctors."* |
| **8. Anchoring change in the organisation's culture:** | *"We are continuously working to maintain it – wehave already scheduled meetings and are considering training. We are also looking at how to integrate it into the onboarding process for new staff."*    *"I believe it has potential. You cannot learn everything from reading. Some things are best learned through shadowing. It really makes a difference because we are dealing with such complex patients. We cannot capture it all in a manual."* |
